# Identification of five hub immune genes and characterization of two immune subtypes of osteoarthritis

**DOI:** 10.3389/fendo.2023.1144258

**Published:** 2023-03-16

**Authors:** Lifeng Pan, Feng Yang, Xianhua Cao, Hongchang Zhao, Jian Li, Jinxi Zhang, Jiandong Guo, Zhijiang Jin, Zhongning Guan, Feng Zhou

**Affiliations:** ^1^ Department of Orthopaedics, Hangzhou Ninth People’s Hospital, Hangzhou, Zhejiang, China; ^2^ Community Health Service Center, Hangzhou, Zhejiang, China

**Keywords:** osteoarthritis, immune microenvironment, diagnostic model, nomogram, machine learning

## Abstract

**Background:**

Osteoarthritis (OA) is one of the most prevalent chronic diseases, leading to degeneration of joints, chronic pain, and disability in the elderly. Little is known about the role of immune-related genes (IRGs) and immune cells in OA.

**Method:**

Hub IRGs of OA were identified by differential expression analysis and filtered by three machine learning strategies, including random forest (RF), least absolute shrinkage and selection operator (LASSO), and support vector machine (SVM). A diagnostic nomogram model was then constructed by using these hub IRGs, with receiver operating characteristic (ROC) curve, decision curve analysis (DCA), and clinical impact curve analysis (CICA) estimating its performance and clinical impact. Hierarchical clustering analysis was then conducted by setting the hub IRGs as input information. Differences in immune cell infiltration and activities of immune pathways were revealed between different immune subtypes.

**Result:**

Five hub IRGs of OA were identified, including TNFSF11, SCD1, PGF, EDNRB, and IL1R1. Of them, TNFSF11 and SCD1 contributed the most to the diagnostic nomogram model with area under the curve (AUC) values of 0.904 and 0.864, respectively. Two immune subtypes were characterized. The immune over-activated subtype showed excessively activated cellular immunity with a higher proportion of activated B cells and activated CD8 T cells. The two phenotypes were also seen in two validation cohorts.

**Conclusion:**

The present study comprehensively investigated the role of immune genes and immune cells in OA. Five hub IRGs and two immune subtypes were identified. These findings will provide novel insights into the diagnosis and treatment of OA.

## 1 Introduction

Osteoarthritis (OA) is one of the most prevalent chronic diseases worldwide, leading to degeneration of joints, chronic pain, and disability in the elderly (1). Novel insights suggested that OA is a syndrome of joint destruction caused by different risk factors, and each of the factors could promote OA by instigating different mechanistic pathways (2). Typical processes involved in OA development contain mechanical (3), inflammatory (4), metabolic (5), and senescent (6) signaling pathways. Interestingly, synovitis is found in the majority of patients with OA. Moreover, the infiltration of T cells and activated macrophages in synovial tissue has a strong correlation with bone erosion and pain in OA patients (7). Little is known, however, about the osteo-immune microenvironment (OIME) of OA, and the role of immune-related genes has hardly been studied in this disease.

Hereby, we investigated the role of immune-related genes (IRGs) in OA from the aspects of OIME, disease classification, and diagnostic value. First, hub IRGs were identified by differential expression analysis and three strategies of feature selection, including random forest (RF), least absolute shrinkage and selection operator (LASSO), and support vector machine (SVM). Then, these hub IRGs were used to construct a diagnostic nomogram model with receiver operating characteristic (ROC) curve, decision curve analysis (DCA), and clinical impact curve analysis (CICA) estimating its diagnostic performance and clinical impact for OA. These hub IRGs were then subjected to hierarchical clustering analysis, and two immune subtypes of OA were characterized. The immune over-activated subtype showed a higher proportion of activated B-cell and activated CD8 T-cell infiltration, underlying an OIME with excessively activated cellular immunity for this group. Finally, two external cohorts of OA were utilized to validate the existence of the two immune subtypes of OA.

In all, the present study conducted a comprehensive analysis of the role of immune genes and immune cells in OA. An immune over-activated subtype of OA was identified, and a nomogram model was built for clinical practice. It was found that regulatory T-cell infiltration was positively correlated with TNFSF11 and IL1R1 and negatively correlated with EDNRB. These findings provided novel insights to understand the role of the osteo-immune microenvironment in the development of OA.

## 2 Materials and methods

### 2.1 Data collection and processing

The microarray datasets were downloaded from the Gene Expression Omnibus (GEO) database (https://www.ncbi.nlm.nih.gov/geo/) using “Osteoarthritis”, “Tissue”, and “Homo sapiens” as keywords. The microarray datasets GSE55235 and GSE55457 (doi: 10.1186/ar4526) and GSE82107 (doi: 10.1371/journal.pone.0167076) contained 27 healthy controls and 30 OA patients. A dataset of identified IRGs was acquired from the ImmPort database (http://www.immport.org).

We then performed log2 transformed for gene expression profiling and matched the probes to their gene symbols according to the annotation document of corresponding platforms. Finally, the gene matrix with row names as sample names and column names as gene symbols were obtained for subsequent analyses.

### 2.2 Identification of differentially expressed immune-related genes

These three datasets were merged and normalized by the “limma” package8 of R software (doi: 10.1093/nar/gkv007). The batch effect amid different arrays was eliminated by using the ComBat function of R (version 4.1.3) package sva9. We extracted the expression profiles of immune-related genes from this merged dataset. Then, we identified differentially expressed IRGs in OA and normal samples by the “limma” package. p-value <0.05 was considered a significant difference. Heatmap was generated using the R package “pheatmap” to visualize the differentially expressed IRGs.

### 2.3 Functional and pathway enrichment analyses

To investigate the functional and molecular pathways of differentially expressed IRGs, we performed Gene Ontology (GO) (8), Kyoto Encyclopedia of Genes and Genomes (KEGG) (9), and gene set enrichment analysis (GSEA) (10) enrichment analyses by the “colorspace”, “stringi”, and “ggplot2” packages in R (doi: 10.7717/peerj.11534). p < 0.05 was considered statistically significant.

### 2.4 Screening of OA-related biomarker characteristic genes

The protein–protein interaction (PPI) network was constructed to predict protein–protein interactions of differentially expressed IRGs using the Search Tool for the Retrieval of Interacting Genes database (STRING, http://www.stringdb.org). The gene with an interaction score >0.9 was retained, and Cytoscape software v3.6.0 is used to visualize the PPI network. Based on these IRGs, three feature selection algorithms including SVM–recursive feature elimination (SVM-RFE), LASSO logistic regression, and RF were adapted to screen OA-related biomarkers. The SVM-RFE algorithm was performed by R packages “e1071” and “caret” with fivefold cross-validation. The LASSO logistic regression was employed with the R package “glmnet” (11). The RF algorithm was analyzed by the “randomForest” package in R (https://CRAN.R-project.org/package=beeswarm). Then, the “venn” R package (12) (version 1.7) was used to select overlapping genes from the three algorithms as signature genes for further analysis.

### 2.5 Construction of a nomogram model

The ROC and area under the curve (AUC) were also calculated to evaluate the predictive effectiveness of the algorithm. We constructed a nomogram model based on OA-related signature genes to predict the occurrence of OA patients with the “rms” package in R. The calibration curve was used to assess the predictive performance of the nomogram model. Then, we further performed DCA and CICA to estimate the clinical utility of the nomogram model.

### 2.6 Consensus clustering

Consensus clustering is an algorithm for identifying the cluster of each member and their number in datasets. We utilized the consensus clustering method to distinguish distinct immune-related clinical subtypes of OA and identify different IRG patterns based on the significant differentially expressed IRGs with the R package “ConsensusClusterPlus” (13). In the correlation between significant OA-related IRG expression and clinical features in subtypes of OA patients. “Points” represents the score of the corresponding factor below, and “Total Points” indicates the summation of all the scores of factors above.

### 2.7 Estimation of immune cell infiltration

The single-sample gene set enrichment analysis (ssGSEA) was employed to measure the relative abundance of immune cells in OA samples *via* the R packages “limma”, “GSVA” (10), and “GSEABase”. The gene set for marking each immune cell type was obtained from the study of Charoentong (14). We also conducted a correlation analysis of immune cells with OA-related genes.

### 2.8 Calculation of immune score

We used principal component analysis (PCA) algorithms to construct the signature of immune-related genes for OA samples (doi: 10.1038/nbt0308-303). Principal Component 1 (PC1) and Principal Component 2 (PC2) were chosen as the signature scores. Immune scores for each OA patient were calculated using the formula Immune Score = Σ(PC1i + PC2i), where i is the expression of immune-related genes. We calculated the relationship between different classifications and immune scores. We used limma and ggpubr packages to study the relationship between the different classifications and the expression level of notable molecules.

### 2.9 Statistics and software

Data processing and bioinformatics analyses were accomplished by R (version 4.1.3). Packages limma, ggplot2, rmda, clusterProfiler, ssGSEA, rsm, and glmnet were employed for analyses with proper citations. The Wilcoxon or Kruskal–Wallis test was applied for comparisons between two or more groups involved in this study. Pearson’s and Spearman’s rank correlation tests were adopted to estimate the statistical correlation of parametric or non-parametric variables. Two-sided p < 0.05 was considered a significant threshold for all statistical tests.

## 3 Results

### 3.1 Hub IRGs and their biological function in OA

**Figure 1:**
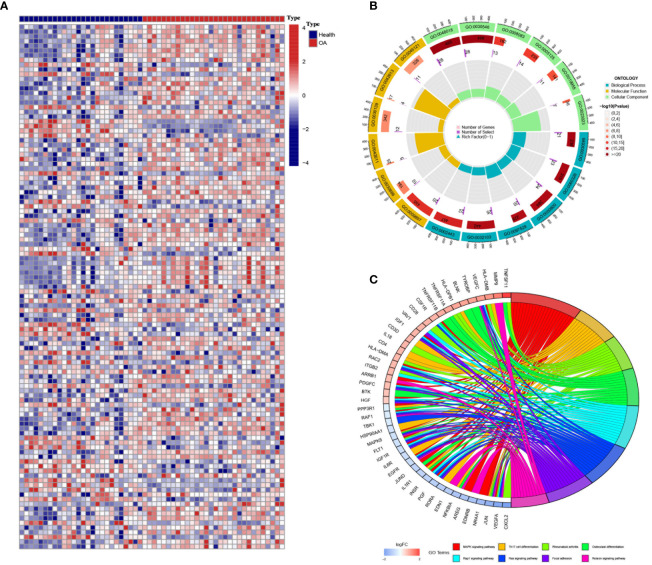
Differentially expressed immune genes in osteoarthritis (OA). **(A)** The heatmap shows the differentially expressed immune genes between OA and control samples (GSE55235). **(B, C)** Gene Ontology **(B)** and Kyoto Encyclopedia of Genes and Genomes **(C)** enrichment analyses revealed the biological function and downstream pathways of the differentially expressed immune genes.

Between the OA samples and the control samples, there was a significant difference in the expression of 2,483 IRGs (**Figure 1A**). As was to be predicted, enrichment of these genes was found in a number of processes related to bone production and resorption. These processes include MAPK, Osteoclast Differentiation, and Ras Signaling Pathways. In addition to this, the Th17 cell differentiation pathway was shown to be active in OA patients, which suggests the possible involvement of immune cells in the development of OA (**Figures 1B, C**).

### 3.2 Diagnostic value of the hub IRGs in OA

**Figure 2:**
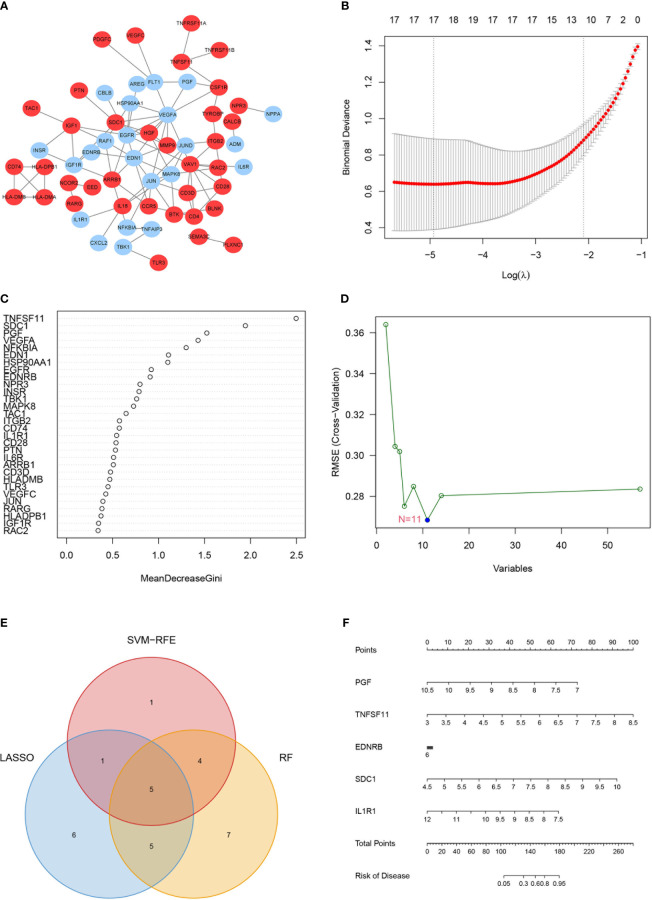
Hub immune-related genes (IRGs) and their diagnostic value. **(A)** Protein–protein interaction network of the IRGs. **(B–D)** Hub IRGs were filtered by three machine learning strategies of feature selection, including least absolute shrinkage and selection operator **(B)**, random forest **(C)**, and support vector machine **(D)**. **(E)** Five hub IRGs were identified by the three machine learning strategies. **(F)** The five-IRG-based nomogram model showed good diagnostic performance.

There were intense interactions amid these IRGs, and several genes seemed to be key regulators in OA, including VEGFA, EDN1, JUN, and MAPK8 (**Figure 2A**, **Figure S1**). Three machine learning strategies were then utilized for feature selection by inputting these IRGs and patients’ diagnostic information (**Figures 2B-D**). Finally, 17, 11, and 21 core genes were authenticated by LASSO, SVM, and RF algorithms, respectively (**Figure 2E**). Of them, five intersected genes were submitted to the final diagnostic model, including PGF, TNFSF11, EDNRB, SDC1, and IL1R1 (**Figure 2E**).

In the end, TNFSF11 and SDC1 appeared to contribute the most in the diagnostic model to distinguish OA samples from control samples, suggesting that these two genes play an important role in the progression of OA (**Figure S2**). The AUC for TNFSF11 was 0.904 (0.806–0.979), and the AUC for SDC1 was 0.864 (0.744–0.959) (**Figures S2D,E**). The nomogram then quantified the contribution of each gene, and as a result, the patients’ disease risk was quickly calculated by adding up the points from all five genes (**Figure 2F**). In the calibration curve, the nomogram’s predicted disease risk and the actual disease condition were quite congruent with one another (**Figure S3A**). The subsequent DCA study demonstrated a significant internal advantage for this approach (**Figure S3B**). When the value of the threshold was greater than 0.6, the estimated number of patients came closer to matching the actual positive patient count (**Figure S3C**).

### 3.3 Characterization of the immune over-activated and immune-inhibited subtypes of OA

**Figure 3:**
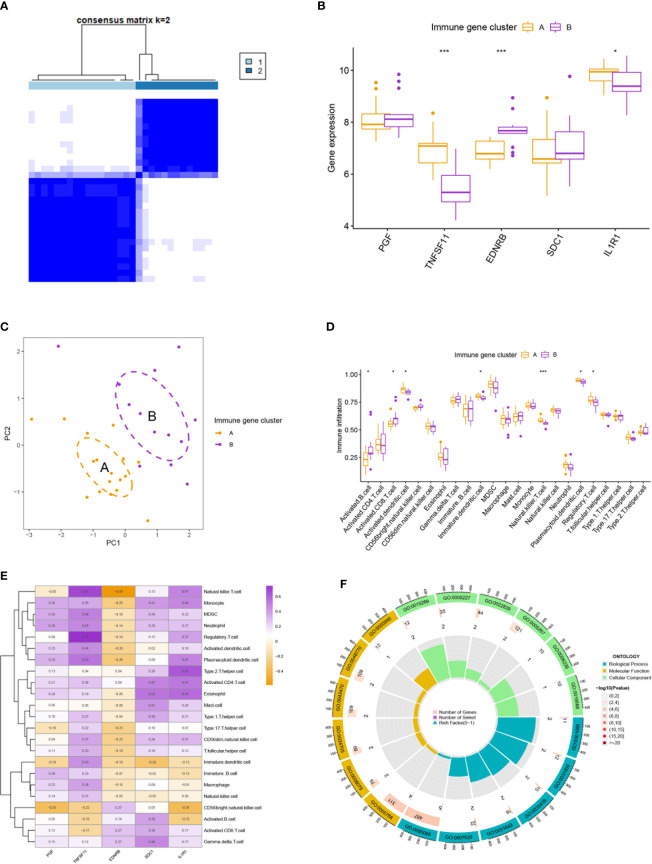
Clustering analysis and immune infiltration analysis. **(A)** Clustering analysis stratified patients into two subtypes. **(B, C)** The two immune subtypes differ in gene expression pattern **(B)** and geometrical distance **(C)**. **(D)** Immune subtype B showed higher infiltration of activated B cells and activated T cells than subtype **(A)**. **(E)** Correlation analysis between five hub immune genes and immune cells. **(F)** Gene Ontology enrichment analysis revealed the functional differences between the two immune subtypes. * means P < 0.05, *** means P < 0.001.

Two subtypes of OA were identified by executing hierarchical clustering analysis with the IRGs mentioned above (**Figures 3A, C**). Cluster A displayed higher expression of TNFSF11 and IL1R1, while Cluster B demonstrated an increased level of EDNRB (**Figure 3B**). Moreover, Cluster B was seen with increased infiltration of activated B cells and activated CD8 T cells and decreased infiltration of regulatory T cells, suggesting a microenvironment with excessively activated cellular immunity for this group (**Figure 3D**). On the contrary, Cluster A seemed to be the immune-inhibited subtype of OA with more infiltration of regulatory T cells. Correspondingly, TNFSF11 and IL1R1 were found positively correlated with the infiltration of regulatory T cells, partly accounting for its reduction in Cluster B (**Figure 3E**). In addition, Clusters A and B differed in many biological processes (**Figure 3F**) such as regulation of anatomical structure size (go:0090066), endoplasmic reticulum lumen (go:0005788), potassium channel activity (go:0005267), and heat generation (go:0031649).

### 3.4 External validation for the two immune subtypes in GSE55457 and GSE82107

**Figure 4:**
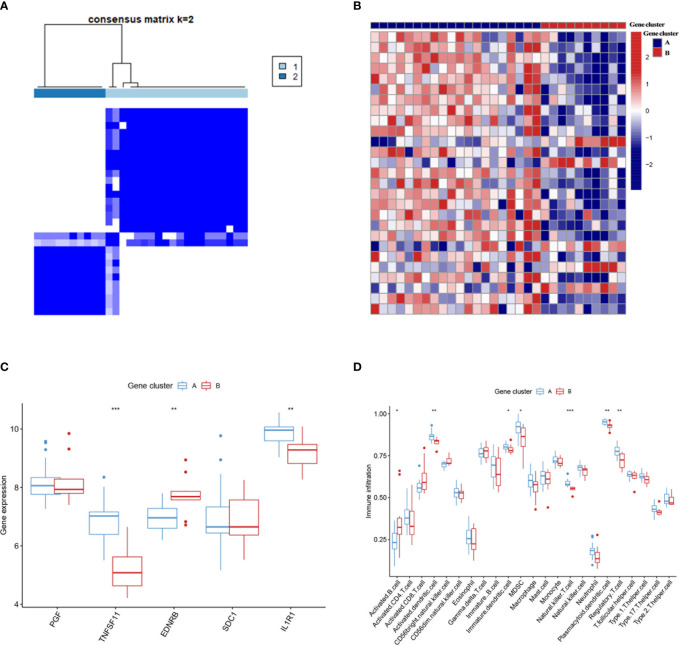
External validation for the two immune subtypes in GSE55457. **(A)** Two immune subtypes were found in GSE55457 by clustering analysis. **(B)** The heatmap showed the differentially expressed genes between the two subtypes. **(C, D)** The two immune subtypes differ in the pattern of immune gene expression **(C)** and immune cell infiltration **(D)**. Cluster B also displayed higher infiltration of activated B cells and T cells as the subtype B in GSE55235. * means P < 0.05, ** means P < 0.01, *** means P < 0.001.

**Figure 5:**
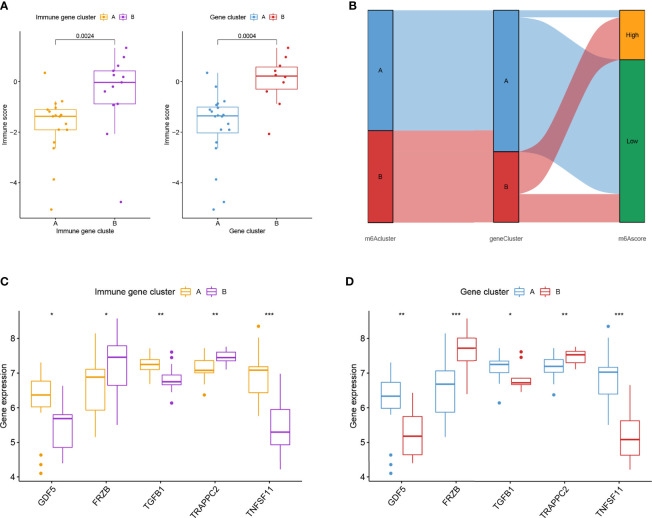
External validation for the two immune subtypes in GSE82107. **(A)** Boxplot showed the difference in immune score in the immune gene cluster and gene cluster in GSE82107. **(B)** The Sankey diagram showed the distribution of patients in different clusters. **(C, D)** Expression difference of five osteoarthritis-related genes in the immune gene cluster **(C)** and gene cluster **(D)**. * means P < 0.05, ** means P < 0.01, *** means P < 0.001.

Similar classifications were seen in two external validation cohorts: GSE55457 (N = 33) and GSE82107 (N = 17). The processes of clustering analyses for these two cohorts were illustrated in supplementary pictures (**Figures S4**, **S5**) with consensus matrix, CDF, and delta area determining the optimal number of clusters. Distinguishable two clusters were identified in GSE55457 with a group of genes upregulated in Cluster A (**Figures 4A, B**). Keeping consistent with the former results of the training cohort, TNFSF11, IL1R1, and regulatory T cells also showed a marked decrease in Cluster B (**Figures 4C, D**), implying a phenotype of immune over-activation with advanced bone absorption. In GSE55457, Cluster B was seen with an increased immune score in both the immune gene cluster and the gene cluster, supporting the immune-activated phenotype of this group. The Sankey diagram demonstrated the overlap of patients between the different clusters (**Figures 5A, B**). In parallel, Cluster B showed a distinct decline of TNFSF11 and GDF5, accompanied by significant ascending of FRZB and TRAPPC2 (**Figures 5C, D**).

## 4 Discussion

Non-infectious chronic inflammation, which occurs when inflammatory cells invade synovial tissue or synovial fluid, especially in the early stages of the illness, is the main clinical hallmark of OA (doi: 10.1053/joca.1998.0224, 10.1002/art.10768). Immunity plays a key role in the emergence and progression of OA.

The present study comprehensively investigated the role of immune genes and immune cells in OA, revealing the immune over-activated and immune-inhibited subtypes of OA. The former subtype showed higher infiltration of activated B cells and CD8 T cell, compared with lesser infiltration of regulatory T cells, underlying a microenvironment with excessive cellular immunity. A nomogram model was also constructed by using five immune genes, showing rather good diagnostic performance. These findings will help understand the crosstalk between immune cells and bone tissue, providing novel insights for the diagnosis and treatment of OA.

First, five critical IRGs were identified in our study, including PGF, TNFSF11, EDNRB, SDC1, and IL1R1. It was shown that the presence of regulatory T cells was inversely connected with EDNRB and positively correlated with TNFSF11 and IL1R1. Of them, TNFSF11 contributed most significantly to the diagnosis of OA, followed by SCD1. Reportedly, TNFSF11 (TNF Superfamily Member 11) is a key factor responsible for osteoclast differentiation and activation, encoding RANKL, the ligand of osteoprotegerin (OPG) (15, 16), to regulate bone density. Moreover, TNFSF11 has already been linked to a series of diseases with osteoproliferation or osteolysis, including osteopetrosis, dysosteosclerosis, Paget disease of bone 2, and familial expansile osteolysis (17). Therefore, it is reasonable to see the significant upregulation of TNFSF11 in osteoarthritis. Correspondingly, reducing TNFSF11 expression could relieve the progression of cartilage degradation in OA (18).

Meanwhile, TNFSF11 is key in the processes of lymph node development and production of activated B cells and T cells (19, 20). This is consistent with the results of our study: TNFSF11 was observed to be correlated with the infiltration of activated T cells, B cells, natural killer T cells, neutrophils, monocytes, etc. Similarly, elevated TNFSF11 was reported to induce a pro-inflammatory phenotype of OA (21), resulting in accelerated joint destruction and deteriorated clinical symptoms (22).

SCD1, stearoyl CoA desaturase 1, was found to promote the function of osteogenesis in bone marrow mesenchymal stem cells (23), and inhibition of SCD1 could prevent postmenopausal osteoporosis to some extent (24). Keeping consistent with these studies, we found that SCD1 also played a pivotal role in OA. SCD1 expression was positively correlated with the infiltration of monocyte, activated CD4 T cell, and gamma delta T cell, underlying an inflamed microenvironment. Potential mechanisms accounting for this correlation between SCD1 and immune imbalance are the activation of miR-203a/FOS and miR-1908/EXO1 signaling pathways by SCD1 (25).

The present study has several advantages. Comprehensive investigations were conducted on the role of immune genes and immune cells in OA. Several critical immune genes were identified, and a diagnostic nomogram was constructed with quite good performance. Immune over-activated and immune-inhibited subtypes of OA were revealed. The former subtype showed higher infiltration of activated B cells and CD8 T cells, underlying a microenvironment with excessive cellular immunity. These findings will provide novel insights into the diagnosis and treatment of OA.

There were also some limitations to our study. First, it would be more convincing if there were some *in vitro* experiments. Second, the expression of TNFSF11, SCD1, and the two immune subtypes of OA could be tested in actual patient cohorts. Lastly, analysis of the pathways related to osteogenesis can be added to further explain the difference between the two immune subtypes of OA.

## Data availability statement

The original contributions presented in the study are included in the article/**Supplementary Material**. Further inquiries can be directed to the corresponding authors.

## Author contributions

FZ, ZG, and ZJ designed the study. LP, FY, XC, HZ, JL, JZ, and JG performed data analysis. LP and FY drafted the manuscript. FZ, ZG, and ZJ revised the manuscript. All authors read and approved the final manuscript.
