# Supplementary figures and images for: Identification of five hub immune genes and characterization of two immune subtypes of osteoarthritis
Source: Front Endocrinol (Lausanne). 2023 Mar 16;14:1144258. doi: 10.3389/fendo.2023.1144258 (PMC10060864; doi:10.3389/fendo.2023.1144258)

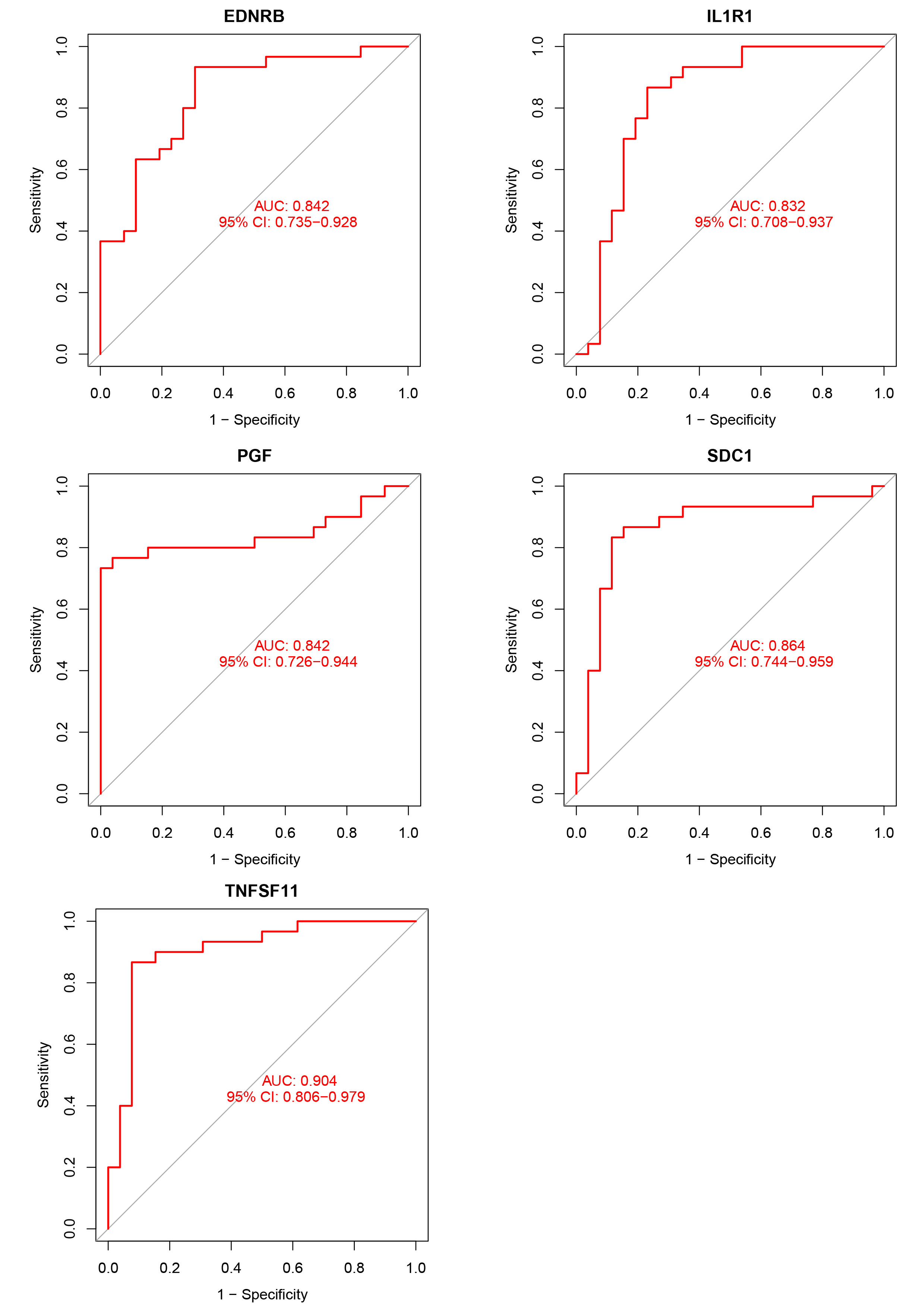

Supplement: Supplementary file 2 [file Image_2.tif]

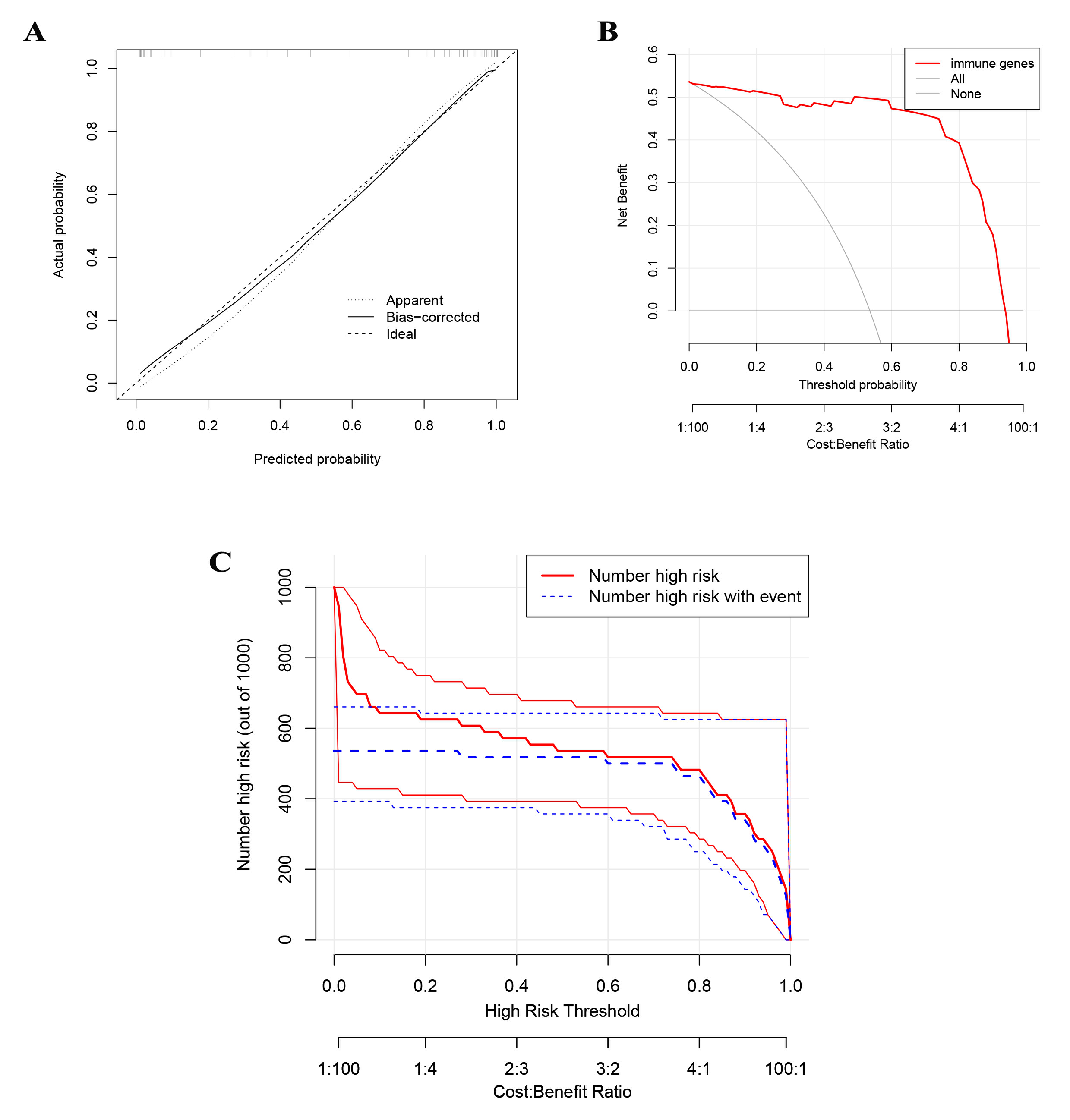

Supplement: Supplementary file 3 [file Image_3.tif]
